# Galactosed and Reduction-Responsive Nanoparticles Assembled from Trimethylchitosan–Camptothecin Conjugates for Enhanced Hepatocellular Carcinoma Therapy

**DOI:** 10.3390/pharmaceutics14071315

**Published:** 2022-06-21

**Authors:** Chen Fu, Jingcan Qin, Xinlong Liu, Fei Kong

**Affiliations:** 1 Department of Pharmacology, School of Pharmacy, China Medical University, Shenyang 110122, China; cfu@cmu.edu.cn; 2 Department of Radiology, Shanghai Jiao Tong University Affiliated Sixth People’s Hospital, Shanghai Jiao Tong University School of Medicine, 600 Yi Shan Road, Shanghai 200233, China; qinjingcan1988@163.com; 3 School of Chemistry and Chemical Engineering, Shanghai Jiao Tong University, 800 Dongchuan Road, Shanghai 200240, China; xinlongliusjtu@sjtu.edu.cn

**Keywords:** galactosed trimethylchitosan, camptothecin, prodrug nanoparticles, hepatocellular carcinoma, targeted delivery, antitumor

## Abstract

The targeted delivery of drugs to tumor cells and prevention of premature release before reaching the target is one of the key challenges to developing nanomedicines. In this paper, galactose decorated trimethyl chitosan (GT)–camptothecin (CPT) prodrug nanoparticles (GT-ss-CPT NPs) were prepared from GT-CPT conjugates linked by dithiodipropionic acid. The obtained GT-ss-CPT NPs were spherical with a particle size of 184.1 nm. GT-ss-CPT NPs displayed low drug release under physiological conditions, whereas efficient drug release was triggered by high GSH concentration. GT-ss-CPT NPs exhibited a higher antitumor effect both in vitro and in vivo than the free drug counterpart. More importantly, GT-ss-CPT NPs reduced the high systematic toxicity of CPT to tumor-bearing mice. In summary, GT-ss-CPT NPs can not only inhibit the premature release of CPT but also have a great potential for targeted hepatocellular carcinoma chemotherapy.

## 1. Introduction

In the past decades, nanomedicine has emerged as an effective strategy for tumor therapy. Many nanoscale systems including prodrugs, liposomes, micelles, nanoparticles, etc. for the targeted delivery of small chemical drugs, proteins, and nucleic acids to treat tumors have been developed and studied. Among these systems, prodrugs and micelles have been extensively studied. They have been shown to overcome the various drawbacks of anticancer drugs, such as poor water solubility and toxic side effects on healthy tissues. However, prodrugs are primarily subject to rapid clearance, whereas micelles often face low drug loading, poor stability, and premature drug leakage [1]. To overcome the shortcomings of prodrugs and micelles, covalently linking prodrugs with nanosized carriers composed of antibodies, proteins, polysaccharides, peptides, or polymers to construct prodrug micelles or nanoparticles has become an alternative strategy and has been widely explored in various tumor therapy studies [2,3].

Hepatocellular carcinoma (HCC), one of the top five deadliest cancers across the world, has a poor survival rate, while the need for new more effective therapy is required [4]. The asialoglycoprotein receptor (ASGPR) is a hepatocyte-specific receptor that is abundantly overexpressed on hepatocytes and minimally on extra-hepatic regions [5]. Hence, ASGPR has been employed as a useful targeting site in the HCC treatment [6,7,8]. Chitosan, a naturally nontoxic polymer with excellent biodegradability and biocompatibility, has been widely used as a carrier for anticancer drug delivery. However, chitosan suffers from its poor solubility and a lack of tumor-targeting ability. Currently, different modifications of chitosan such as ligand modification have been made for addressing the issues faced by chitosan for tumor-targeted drug delivery [9,10]. Galactosylated chitosan or chitosan derivatives are potential carriers that can effectively target anticancer drugs to improve therapeutic effects for HCC by virtue of the galactose residue specifically recognized by ASGPR. Huang et al. fabricated galactosed-chitosan-5-fluorouracil acetic acid conjugate-based nanoparticles, which could specifically recognize ASGPR receptors on HCC cell surface, and these generated higher antitumor efficacy than free drugs in both in vitro and in vivo studies [11]. The galactosed trimethyl chitosan (GT) derivative that overcomes the defects of chitosan as a delivery carrier, e.g., poor water solubility and tumor targeting, has been proved to be an effective gene carrier for the targeted treatment of HCC [12,13]. However, GT as a carrier in the construction of prodrugs for the targeted treatment of HCC has been rarely studied yet.

Camptothecin (CPT) is a cytotoxic, quinoline alkaloid drug, which possesses superior antitumor efficacy, antiproliferative effect, and apoptosis induction capacity against a wide range of tumors. It has been demonstrated that CPT can inhibit the growth, invasion, metastasis, and angiogenesis of HCC both in vivo and in vitro by down-regulating the nuclear factor E2-related factor 2 (Nrf2) [14]. However, at physiologic pH, the lactone ring of CPT (active form) can be easily hydrolyzed to the open-ring hydroxy acid (inactive form), which results in a less potent antitumor activity but is accompanied by severe toxicity [15,16]. Thus, the main obstacles associated with tumor therapy are its intrinsic high toxicity, poor water solubility, and stability [15]. Currently, prodrug nanoscale delivery systems are being applied to overcome these obstacles, and several CPT-based prodrug nanoscale delivery systems have entered the clinical practice so far [17]. The triggered release of bioactive drugs in target cells or tissues from prodrugs can reduce high systemic toxicity. This strategy can be achieved via linkers in response to various endogenous stimuli such as low pH, high glutathione (GSH) concentrations, and specific enzymes, as well as exogenous stimuli, such as heat and light [3,18]. In particular, disulfide linkages have been widely exploited for tumor drug delivery because of a specific degradation in response to elevated GSH concentration in tumor cells through a thiol–disulfide exchange reaction [19]. Recently, two GSH-responsive nanoparticle drug delivery systems based on disulfide bond linked lactose-S-S-CPT prodrugs were prepared for targeted HCC therapy. Due to the distinctly different levels of GSH in tumor cells and physiological conditions, these CPT-prodrug nanoparticles could remain stable under physiological conditions but rapidly release active drugs after uptake by HCC cells with ASGPR-mediated endocytosis, which obtained better HCC curative performances [8,20].

In this paper, CPT prodrug nanoparticles were prepared from GT-CPT conjugates linked by dithiodipropionic acid (GT-ss-CPT NPs). The GT-ss-CPT NPs via disulfide linkers were supposed to release the active CPT in response to intracellular GSH. GT was proposed to act as an effective carrier for CPT to targeted HCC delivery and therapy. The physicochemical properties and in vitro drug release behavior of GT-ss-CPT NPs were investigated. More importantly, in vitro and in vivo antitumor effects and safety were studied.

## 2. Materials and Methods

### 2.1. Materials, Cell Lines, and Animals

Chitosan (*Mw* of 150 kDa, 85% deacetylated degree) was purchased from Goldenshell Biotechnology Co., Ltd. (Taizhou, China). Dithiodipropionic acid, lactobionic acid (LA), galactose, 3-(4,5-dimethyl-2-thiazolyl)-2,5-diphenyl-2-H-tetrazolium bromide (MTT), and CPT were purchased from Chemiejoy Inc. (Shanghai, China). 1-(3-Dimethylaminopropyl)-3-ethylcarbodiimide hydrochloride (EDC), N-Hydroxysuccinimide (NHS), Nile red, and dry dimethylsulfoxide (DMSO) were bought from Meryer (Shanghai) Chemical Technology Co., Ltd. (Shanghai, China). FITC-Annexin V/propidium iodide (PI) and TdT-mediated dUTP Nick-End Labeling (TUNEL) apoptosis detection kits were purchased from Yeasen Biotechnology (Shanghai) Co., Ltd. (Shanghai, China). A human liver cancer cell line (HepG2), a human cervical cancer line (Hela), and a murine liver cancer cell line (H22) provided by the Chinese Academy of Sciences (Shanghai, China) were cultured in Dulbecco’s modified Eagle’s medium (DMEM, Gibco, Grand Island, NY, USA) containing 10% (*v*/*v*) Fetal Bovine Serum (FBS, Gibco, Grand Island, NY, USA), 100 units/mL penicillin, and 0.1 mg/mL streptomycin (Gibco, Grand Island, NY, USA) at 37 °C in 5% CO_2_. Female Kunming mice (20 ± 2 g) were provided by Jiesijie Experimental Animal Co., Ltd. (Shanghai, China). All animal experiments were approved by the Institutional Animal Care and Use Committee of Shanghai Jiao Tong University and carried out following the Guidelines for Care and Use of Laboratory Animals (Shanghai Jiao Tong University, China).

### 2.2. Synthesis and Characterization of GT-ss-CPT Conjugate

To synthesize the GT-ss-CPT conjugate, GT and a carboxyl-terminated CPT containing disulfide group (COOH-ss-CPT) were synthesized according to previous studies, respectively [12,21,22]. Thereafter, COOH-ss-CPT was covalently grafted to the amino group of GT via EDC/NHS catalysis. Briefly, 0.1 g of CPT-ss-COOH was dissolved in 10 mL of dry DMSO, to which 0.1 g of NHS and 0.2 g of EDC were added. After reaction for 2 h, 10 mL of GT solution (10 mg/mL) was added. Then, the mixture was allowed to react for 48 h at room temperature under a nitrogen atmosphere. Afterward, the DMSO and the excess reactants and by-products were removed from the reaction mixture by extensive dialysis (MWCO 3500 Da) against 50% alcohol solution followed by distilled water. The GT-ss-CPT conjugate was obtained following lyophilization and stored at −20 °C. The CPT, GT, and GT-ss-CPT were characterized using an infrared (IR) spectroscope (Nicolet 6700, Thermo Fisher, USA) and a nuclear magnetic resonance (^1^H NMR) spectrometer (500 MHz, Bruker, Germany). Their ultraviolet-visible (UV-vis) absorption spectroscopies were recorded using UV-vis absorption spectroscopy. The grafting degree of CPT in the GT-ss-CPT conjugate was calculated as the ratio of aromatic group protons (δ (ppm) = 7.2–8.2 ppm) in CPT to acetyl methyl protons (δ (ppm) = 2.0 ppm) in GT as measured by ^1^H NMR.

### 2.3. Preparation and Characterization of GT-ss-CPT NPs

To prepare GT-ss-CPT NPs, 5 mg of GT-ss-CPT conjugate was added to 1 mL of distilled water and subjected to ultrasonication for 10 min. Then, the mean particle size, polydispersity index, and zeta potential of GT-ss-CPT NPs were measured by dynamic light scattering (DLS) using a Malvern Zetasizer Nano ZS (Malvern Instruments, Malvern, UK). The morphology was characterized under a Transmission Electron Microscope (TEM, JEOL2100F, JEOL, Tokyo, Japan). The sample was prepared by drop casting GT-ss-CPT NPs solutions (0.2 mg/mL) onto carbon-coated copper grids; then, it was dried at room temperature and observed at a voltage of 120 kV. The critical aggregation concentration (CAC) was determined with Nile red as a fluorescent probe according to an earlier study [23]. Briefly, 10 μL of Nile red solutions in methanol (2.0 × 10^−6^ mg/mL) was added to 2.0 mL tubes, and the solvent was allowed to evaporate. Then, GT-ss-CPT NPs solutions with a range of concentrations from 1.0 × 10^−4^ to 5.0 × 10^−1^ mg/mL were added. After the sonication for one hour, the fluorescence intensity at 580 nm (excited at 480 nm) was recorded on a Synergy H4 microreader (BioTeK, Winooski, VT, USA), and the logarithmic concentration of GT-ss-CPT NPs curve was drawn. The intersection of the two linear portions of the curve was considered the CAC value.

### 2.4. In Vitro Drug Release

The release of CPT from GT-ss-CPT NPs was studied in different conditions with various concentrations of GSH or different pH values. One milliliter of GT-ss-CPT NPs (1 mg/mL) was added into a dialysis bag (MWCO 3500 Da). The dialysis bag was then soaked in 100 mL (V_0_) of 0.1 M phosphate buffer solution (PBS, pH 7.4 (containing 0 mM, 5 µM, or 10 mM GSH) or pH 6.5 (0 mM GSH), 1% Tween 80 (*w*/*w*)). At predetermined time intervals, 2 mL (V_t_) of release media was taken out and replaced with an equal volume of fresh media. The amount of CPT released was determined by a fluorospectrophotometer as reported by Chen et al. [21]. The fluorescence intensity of CPT solutions with known concentrations at the emission wavelength of 430 nm (excited at 365 nm) was recorded to establish the standard curve. The CPT content in the release medium was calculated according to the standard curve, and the cumulative release percentage was obtained. The cumulative drug release percentage (%) was calculated following equation:

(1) $$\mathrm{Release}\;\mathrm{rate}\;(\%)=\frac{C_{n}V_{0}+V_{t}\sum_{1}^{n-1}C_{i}}{m_{\mathrm{CPT}}}\times 100$$

where m_CPT_ represents the amount of CPT in the nanoparticles, while C_n_ and C_i_ represent the concentration of CPT in the nth and the concentration of the receiving solution at the ith sampling, respectively.

### 2.5. In Vitro Cytotoxicity

The in vitro cytotoxicity of GT-ss-CPT NPs was evaluated by MTT assay on HCC HepG2 cells [10]. Briefly, HepG2 cells were cultured in 96-wells tissue culture plates at a density of 1 × 10^4^ cells/well for 24 h. Then, the cells were cultured in media containing free CPT or GT-ss-CPT NPs with final CPT concentrations from 0.01 to 100 μg/mL. Cells cultured in media without treatment were used as control. After incubation for 24 h and 48 h, the cell viability was determined by MTT assay using a microplate reader (Thermofisher, Waltham, MA, USA). Additionally, HepG2 cells were incubated with 10 mM galactose for 30 min followed by free CPT or GT-ss-CPT NPs additions (CPT concentration at 10 μg/mL) and cultured for another 24 h and 48 h. Meanwhile, Hela cells were also incubated with free CPT or GT-ss-CPT NPs (CPT concentration at 10 μg/mL) for 24 h and 48 h. The cell viability was determined to study the influence of galactose conjugation on the in vitro cytotoxicity of GT-ss-CPT NPs. 

### 2.6. Cell Apoptosis

HepG2 cells were cultured in 6-well tissue culture plates at a density of 1 × 10^5^ cells/well for 24 h. Then, the cells were cultured in media containing free CPT or GT-ss-CPT NPs with a final CPT concentration of 10 μg/mL for another 24 h. The attached and floating cells were collected and washed thrice with cool PBS. The cell apoptosis rate was analyzed with flow cytometry after being stained with a FITC-Annexin V/PI apoptosis detection kit. 

### 2.7. Hemolysis

The hemolysis of nanoparticles was analyzed by referring to the literature [24]. One milliliter of fresh citrated blood obtained from mice was diluted with 10 mL of PBS. Mouse blood red cells (BRCs) were collected by centrifugation (225× *g*, 5 min), washed thrice with PBS, and resuspended in 10 mL PBS. Five hundred microliters of BRC suspension were mixed with an equal volume of GT-ss-CPT NPs to final concentrations of 0.01, 0.05, 0.2, 0.5, and 1 mg/mL. After incubating at 37 °C for 1 h, the mixtures were centrifuged at 225× *g* for 5 min, and 200 μL of supernatant was taken out to measure the absorbance at 540 nm using a microplate reader (Synergy H4, Bio Tek, Winooski, VT, USA). RBCs mixed with water and PBS were used as the positive and negative controls, respectively. The hemolysis rate of BRCs was calculated as:

(2) $$\mathrm{hemolysis}\;\mathrm{rate}\;(\%)=\frac{{\mathrm{OD}}_{\mathrm{sample}}-{\mathrm{OD}}_{\mathrm{negative}\;\mathrm{control}}}{{\mathrm{OD}}_{\mathrm{positive}}-{\mathrm{OD}}_{\mathrm{negative}\;\mathrm{control}}}\times 100$$

### 2.8. In Vivo Antitumor Efficacy

The in vivo antitumor efficacy was evaluated in an H22 hepatoma murine model, which was established by our previous study [25]. When the average tumor size reached 100 mm^3^, the mice were randomly divided into three groups (3 mice/group) and injected intravenously with saline, free CPT, or GT-ss-CPT (CPT equivalent dose 10 mg/kg) on days 0, 2, and 4 [26]. The tumor volume was monitored using calipers every other day and calculated as V (mm^3^) = 0.5 × length × width^2^. After 14 days, the mice were sacrificed, and the tumors and major organs were collected. Tumors were weighed for the tumor inhibition ratio (TIR) calculation. The tumors and major organs were fixed in 4% paraformaldehyde, processed routinely into paraffin blocks, and cut into thin sections. The tumor sections were immune-histologically stained on Ki-67 and dyed with the TUNEL apoptosis assay kit to assess the cell proliferation and apoptosis in tumors. Major organs sections were examined by hematoxylin–eosin staining (H&E) staining for histological analysis [27,28].

### 2.9. Statistical Analysis

All data were expressed as mean ± standard deviation. Statistical significance analysis was evaluated using the analysis of variance (ANOVA) test. * *p* values < 0.05 and ** *p* values < 0.01 were considered as significant and very significant differences between two groups, respectively. 

## 3. Results and Discussion

### 3.1. Synthesis and Characterization of GT-ss-CPT Conjugate

**Figure 1:**
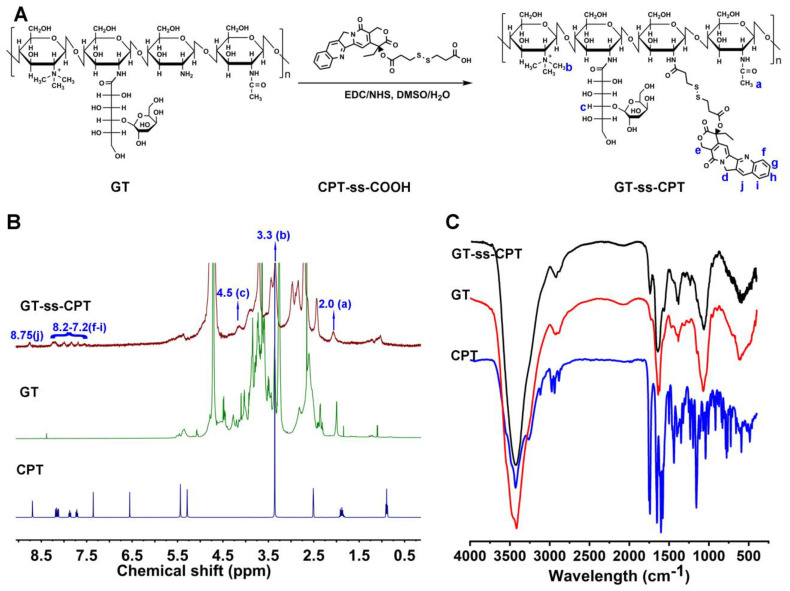
Synthesis and characterization of GT-ss-CPT conjugate. (**A**) Synthetic scheme of GT-ss-CPT conjugate. (**B**) ^1^H NMR spectra and (**C**) FTIR of GT-ss-CPT conjugate, GT, and free CPT.

The synthetic route of the GT-ss-CPT conjugate is shown in Figure 1A. To achieve liver cancer-targeting capacity, LA was firstly conjugated to the trimethyl chitosan. According to the ^1^H NMR spectrum and FTIR spectrum shown in Figure 1B,C, GT was successfully synthesized. The quaternization degree of GT was about 28%, and the modification degree of LA was determined to be about 14.4% by comparing peak areas of the methine groups in LA (4.5 ppm) with the acetamide groups in chitosan (2.0 ppm) [12]. Then, CPT was covalently grafted to the amino group of GT via an amidation reaction in which dithiodipropionic acid was a redox-responsive disulfide bond linker. The successful production of the GT-ss-CPT conjugate was verified by ^1^H NMR (Figure 1B) and FTIR (Figure 1C). As shown in Figure 1B, new proton signals at 7.2–8.2 ppm and 4.5 ppm in the GT-ss-CPT conjugate belonged to the aromatic group proton signals in the CPT and methine groups proton signals in LA, respectively [12,21,29]. The grafting degree of CPT in the GT-ss-CPT conjugate was calculated to be 12.7% (molar ration) by comparing the proton peak areas of CPT (7.2–8.2 ppm) with those of acetamide groups in GT (2.0 ppm). The drug-loading capacity of CPT in the GT-ss-CPT conjugate was 19.1% after conversion from molar ratio to mass ratio. In addition, the characteristic peak of CPT at 1739.79 cm^−1^ (C=O stretch of CPT ester) and characteristic absorption wavelength of CPT at 380 nm were observed in the FTIR spectrum and UV-vis spectrum of the GT-ss-CPT conjugate (Figure 1C, Figure S1), which further confirmed the successful synthesis of this CPT prodrug conjugate [29,30].

### 3.2. Preparation and Characterization of GT-ss-CPT NPs

**Figure 2:**
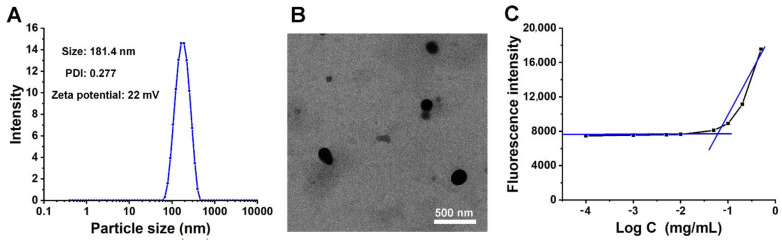
Characterization of GT-ss-CPT NPs. (**A**) Particle size of GT-ss-CPT NPs measured by DLS. (**B**) TEM image showing the morphology of GT-ss-CPT NPs. (**C**) Emission intensity (λ = 580 nm) of Nile red in an aqueous solution of GT-ss-CPT NPs with different concentrations.

As shown in Figure 2A, the particle size of GT-ss-CPT NPs was 184.1 nm with a narrow polydispersity index (PDI, 0.277). The measurements with DLS indicated that GT-ss-CPT NPs possessed a positive charge of 22 mV (Figure 2A). GT-ss-CPT NPs with such a diameter would be favorable for passive targeting to the tumor through the “enhanced permeability and retention” (EPR) effects in addition to the active targeting and result in the efficient accumulation in the tumor. In addition, the positive charge would be able to endorse more cellular attachment and internalization by the tumor cell [31]. The morphology of the GT-ss-CPT NPs as observed by TEM showed approximate spherical shapes (Figure 2C). The particle size as determined by TEM was 100 nm, which was slightly smaller than that observed by DLS. This difference might be due to the dry shrinkage of the sample during preparation for the TEM observation [32]. 

The CAC of GT-ss-CPT NPs was measured using fluorescence spectroscopy with Nile red as a probe [23]. As depicted in Figure 2C, the CAC value of GT-ss-CPT NPs was determined to be 0.061 mg/mL; such a low CAC value would help the GT-ss-CPT NPs maintain stability and nanostructure integrity facing the dilution by a large amount of blood during the blood circulation. 

### 3.3. In Vitro Drug Release

**Figure 3:**
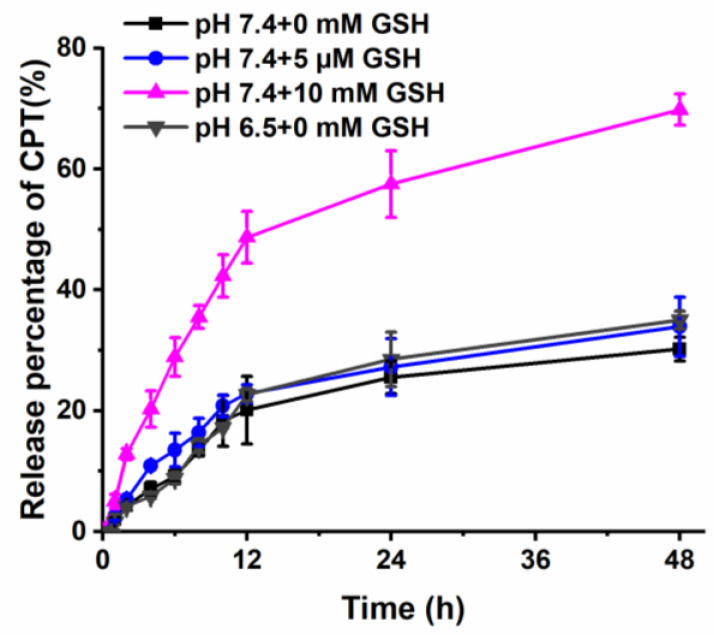
In vitro CPT release profiles from GT-ss-CPT NPs under different GSH concentrations and pH values (*n* = 3).

Disulfide linkages between GT and CPT can be easily broken in the reducing environment. To investigate the reduction responsiveness, the CPT release profile of GT-ss-CPT NPs was recorded in the conditions containing different concentrations of GSH. GSH concentrations of 0 mM, 5 μM, and 10 mM were selected to mimic the reductive microenvironment of blood, extracellular space, and cytoplasm of tumor cells, respectively. As shown in Figure 3, CPT release from GT-ss-CPT NPs was very slow in the medium supplemented with 0 mM GSH, and about 30% of CPT was released within 48 h, which was due to the hydrolysis of ester bonds between CPT-ss-COOH and the GT [33]. As the GSH concentration in the medium increased to 5 µM, CPT release from GT-ss-CPT NPs was slightly increased, but still, less than 34% of CPT was released. In contrast, a significantly faster CPT release from GT-ss-CPT NPs occurred when incubated with 10 mM GSH with about 70% of CPT released within 48 h. Chitosan-based polymers are known for their pH responsiveness. Thus, the release of GT-ss-CPT NPs at pH 6.5 of the tumor microenvironment was also studied. A slightly faster and higher release of CPT from GT-ss-CPT NPs at pH 6.5 was found in comparison to that at pH 7.4, but it was still low (35%), which suggested that GT-ss-CPT NPs have weak pH responsive release behavior. This phenomenon could be due to the increased hydrophilicity of the GT layer and the looseness of GT-ss-CPT NPs as the proton adsorption of GT polymer at pH 6.5, which facilitated the ester bonds cleavage and the release of conjugated drugs [34]. Taken together, these results indicated that GT-ss-CPT NPs are likely to prevent premature drug release in the blood circulation after intravenous injection and in the tumor microenvironment but achieve rapid release CPT in the tumor cells. Therefore, GT-ss-CPT NPs own a high potential for enhancing the therapeutic effects of CPT.

### 3.4. In Vitro Cytotoxicity and Cell Apoptosis

**Figure 4:**
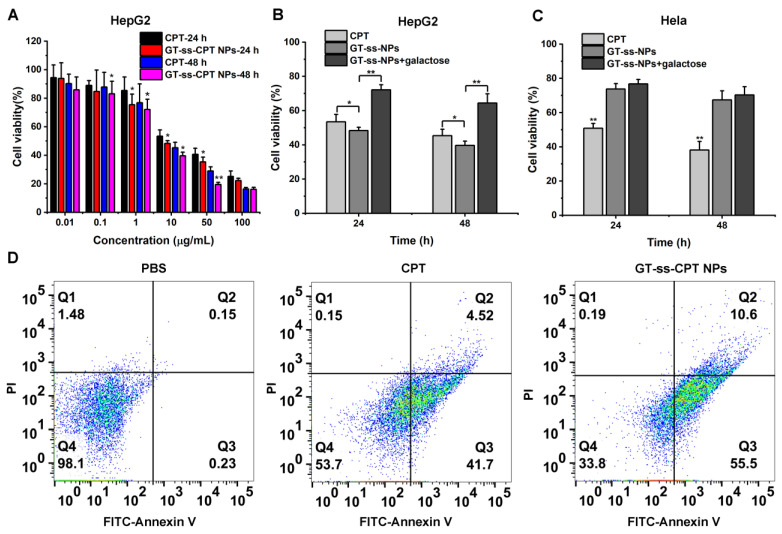
In vitro anticancer activity. (**A**) The cytotoxicity of GT-ss-CPT NPs and free CPT toward HepG2 cells at various concentrations of CPT for 24 h and 48 h incubation (*n* = 6). * *p* < 0.05, ** *p* < 0.01 (ANOVA test). Cell viability of (**B**) HepG2 cells and (**C**) Hela cells incubated with GT-ss-CPT NPs and free CPT for 24 h and 48 h in the presence or absence of galactose (10 mM). (**D**) HepG2 cell apoptosis rate after incubation with PBS, free CPT, and GT-ss-CPT NPs for 24 h. Q1–Q4 quadrants represents necrotic cells, late apoptotic cells, early apoptotic cells, and living cells, respectively.

MTT assay was conducted to investigate the in vitro cytotoxicity of GT-ss-CPT NPs against ASGPR highly expressed HepG2 cells and ASGPR-negative Hela cells compared with free CPT. As shown in Figure 4A, both free CPT and GT-ss-CPT NPs displayed a dose and time-dependent cytotoxicity to HepG2 cells, which were obviously enhanced with the increase in drug concentrations and incubation time. Meanwhile, GT-ss-CPT NPs exhibited higher toxicity toward HepG2 cells than that of CPT as the incubation led to lower cell viabilities. As such, GT-ss-CPT NPs had half-maximal inhibitory concentration (IC_50_) values of 7.93 µg/mL and 5.2 µg/mL against HepG2 cells after incubation for 24 h and 48 h, whereas free CPT had IC_50_ values of 13.7 µg/mL and 8.3 µg/mL, respectively. It can be seen that the IC_50_ values of free CPT were 1.6–1.73 times those of GT-ss-CPT NPs, suggesting that GT-ss-CPT NPs were more effective than free CPT. Moreover, the toxicity of GT-ss-CPT NPs toward HepG2 cells could be greatly inhibited by galactose (Figure 4B). However, lower cytotoxicity of GT-ss-CPT NPs was observed in Hela cells compared with HepG2 cells, and it was not affected by the galactose addition (Figure 4C). This could be due to the lack of ASGPR on the surface of HeLa cells, which makes the nanoparticles unable to be internalized into cells through receptor-mediated endocytosis. These findings revealed that the HCC cells that highly express ASGPR are more susceptible to GT-ss-CPT NPs. To further study the anticancer activity of GT-ss-CPT NPs, cell apoptosis assays were performed using the FITC–Annexin V/PI staining method. As illustrated in Figure 4D, approximately 66.1% of HepG2 cells were apoptotic after incubation with GT-ss-CPT NPs. In contrast, an apoptosis rate of only 46.22% was found in free CPT treated cells, which was much lower than that of GT-ss-CPT cells. Thus, these data demonstrated that grafting CPT to GT to form prodrug nanoparticles could enhance its anticancer activity toward HCC in vitro by targeting the highly abundant ASGPR on the membrane of HCC cells.

### 3.5. Hemolysis

**Figure 5:**
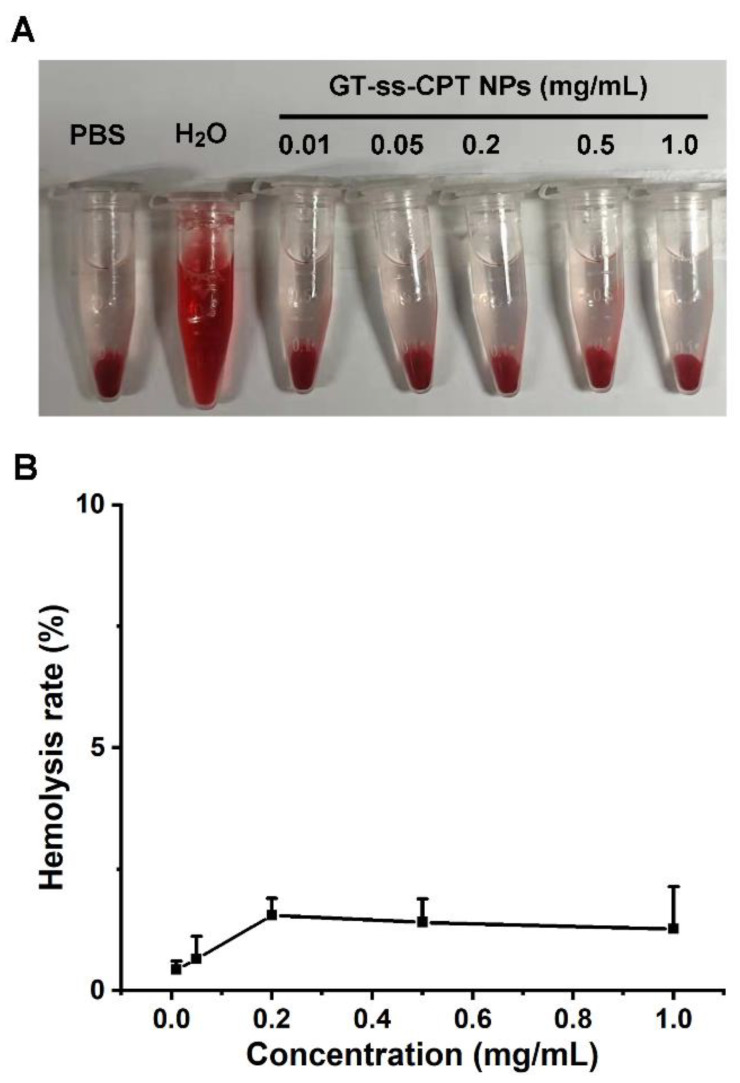
(**A**) Image showing the mice BRCs after incubation with various concentrations of GT-ss-CPT NPs. (**B**) Hemolysis rate of mice BRCs after incubation with various concentrations of GT-ss-CPT NPs (*n* = 3).

In vitro hemolysis testing is often regarded as a simple and reliable indicator for the blood biocompatibility estimation of biomaterials [24,35]. According to the guidance issued by the International Organization for Standardization (ISO 10993-4), biomaterials leading to <5% hemolysis are regarded as safe for blood contacting administration [36]. It could be seen that negligible hemolysis was observed in mouse BRCs after incubation with GT-ss-CPT NPs even at a high concentration of 1.0 mg/mL (Figure 5A). As determined, the hemolysis ratios of GT-ss-CPT NPs at different concentrations were lower than 2% (Figure 5B), which was smaller than the 5% international standard, suggesting these NPs were nonhemolytic. This phenomenon could be ascribed to the decreased cationic density of chitosan after LA and CPT conjugation. Hence, GT-ss-CPT NPs have excellent hemocompatibility suitable for in vivo drug delivery application via intravenous injection. 

### 3.6. In Vivo Antitumor Efficacy

**Figure 6:**
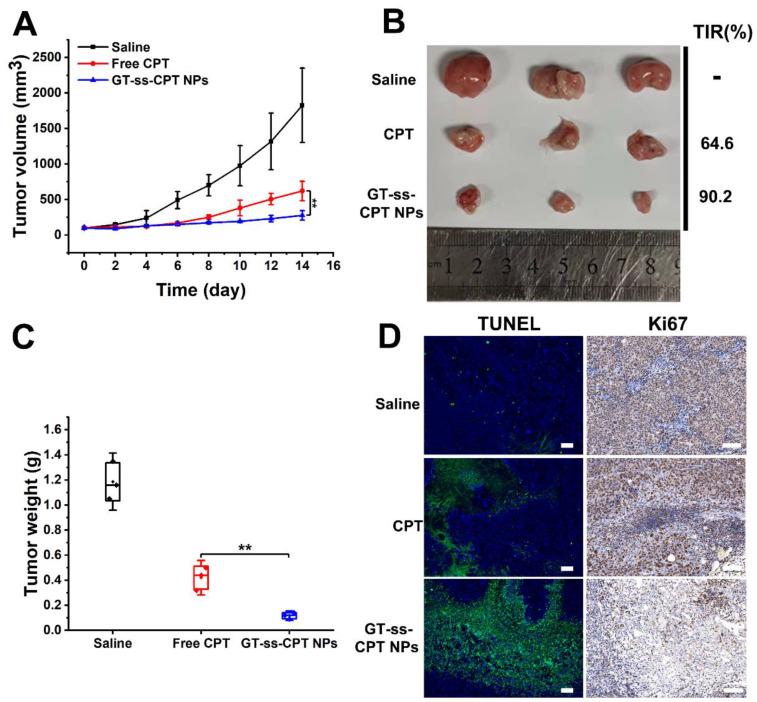
In vivo antitumor effects of GT-ss-CPT NPs against hepatoma carcinoma. (**A**) Tumor volume changes during the therapeutic period (*n* = 3). ** *p* < 0.01 (ANOVA test). (**B**) H22 tumors separated from mice treated with saline, free CPT, and GT-ss-CPT NPs at the end of therapy and the corresponding TIRs. (**C**) Tumor weight from mice treated with saline, free CPT, and GT-ss-CPT NPs at the end of therapy (*n* = 3). ** *p* < 0.01 (ANOVA test). (**D**) Representative TUNEL and Ki-67 immunohistochemical staining of the tumor tissues. Scale bar: 100 µm.

Due to the better tumor cell proliferation inhibitory effect of GT-ss-CPT NPs in vitro compared with free CPT, an H22 hepatoma murine model was established to evaluate the in vivo antitumor efficacy compared with free drugs. As illustrated in Figure 6A,B, the tumor volume of mice injected with saline increased continuously during the experimental period and reached 1825 mm^3^ at the end of the experiment. Both GT-ss-CPT NPs and free CPT showed significant tumor growth inhibitory effects. Compared with tumors in mice injected with free CPT, tumors in mice treated with GT-ss-CPT NPs grew more slowly, leading to a smaller volume at the end of trials. Consistently, tumor weight in the mice injected with GT-ss-CPT NPs was remarkably lower than that of mice treated with free CPT (Figure 6B,C). Thus, the TIR of GT-ss-CPT NPs was significantly higher than that of free CPT (90.2% vs. 64.6%) (Figure 6B). Moreover, Ki67 staining and TUNEL staining were utilized to evaluate in vivo proliferation and in vivo apoptosis. It could be seen from the Ki67-stained tumor sections (Figure 6C) that GT-ss-CPT NPs could suppress the proliferation of H22 cells in vivo more effectively than free CPT. After TUNEL staining (Figure 6C), more cell apoptosis could be observed in tumor sections of mice treated with GT-ss-CPT NPs compared to free CPT. All these results suggested the higher antitumor effects of GT-ss-CPT NPs in vivo than free CPT.

**Figure 7:**
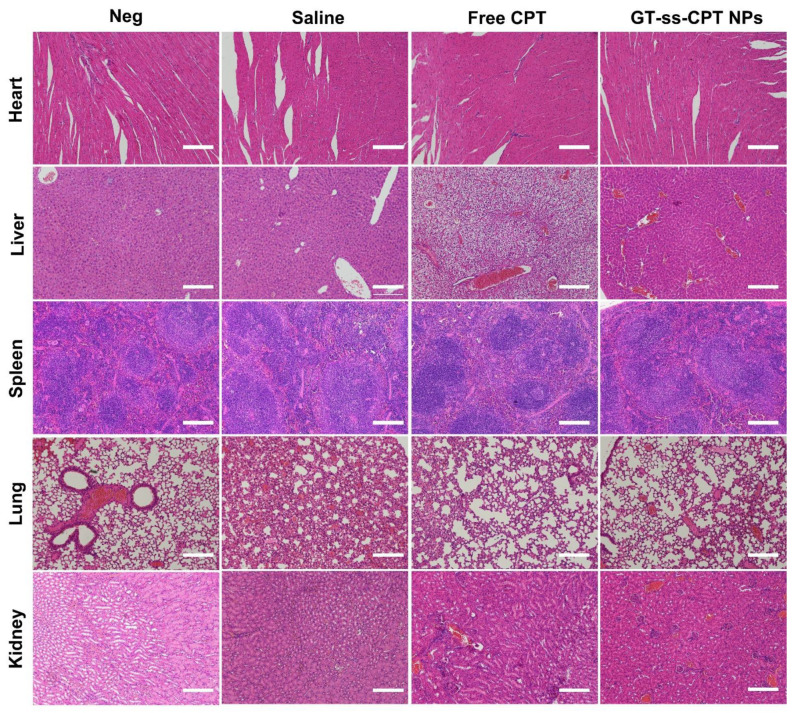
H&E staining images of major organs in H22 tumor-bearing mice treated with saline, free CPT, and GT-ss-CPT NPs at the end of in vivo antitumor trials. Organs from healthy mice were given as negative (Neg) controls. Magnification: 200×.

It is known that as a topoisomerase inhibitor, CPT may cause severe systematic toxicity during antitumor treatment. Thus, histological analysis was performed to investigate the in vivo toxicity of GT-ss-CPT NPs after treatment [27,28]. As seen from the organ sections stained with H&E in Figure 7, no noticeable damages occurred in the heart, liver, spleen, lungs, and kidneys of mice treated with saline. However, significant hepatotoxicity was observed in mice injected with free CPT. Free CPT caused severe necrosis of hepatocytes, loss of liver parenchyma, and absence of sinusoids. An inflammatory response was also observed in the liver, as evidenced by the infiltration of phagocytic cells. This liver toxicity might be due to the systemic elevation of tumor necrosis factor (TNF) levels due to the impaired immune system during CPT treatment, which sensitizes mouse hepatocytes toward TNF-mediated apoptosis [37]. Moreover, slight inflammation and necrosis were found in the heart and kidneys of the mice treated with free CPT, suggesting the toxicity of kidneys and cardiac toxicity of this drug. These results were consistent with the observations from previous studies [38,39]. In contrast, only slight inflammation was found in the liver of mice treated with GT-ss-CPT NPs, and no obvious damage was found in other organs, indicating the safety of GT-ss-CPT NPs. The safety of GT-ss-CPT NPs might be explained by the low drug release under physiological conditions due to their reduction responsiveness, which probably decreased the drug accumulation in normal organs compared with the free drug. To sum up, GT-ss-CPT NPs may be a promising platform for anticancer-targeted drug delivery in liver cancer therapy.

## 4. Conclusions

In summary, galactose decorated trimethyl chitosan–camptothecin conjugates (GT-ss-CPT) were designed and successfully synthesized. GT-ss-CPTs were self-assembled to form prodrug nanoparticles (GT-ss-CPT NPs) with a diameter of 184.1 nm in a spherical shape. GT-ss-CPT NPs displayed low drug release under physiological conditions, whereas efficient drug release was triggered by high GSH concentration. GT-ss-CPT NPs exerted a low hemolysis effect on the mouse BRCs. GT-ss-CPT NPs exhibited an improved antitumor effect both in vitro and in vivo in liver cancer in comparison to the free CPT. More importantly, GT-ss-CPT NPs reduced the high systematic toxicity of CPT. All the results suggested that GT-ss-CPT NPs may be a promising platform for anticancer targeted drug delivery in liver cancer therapy.

## Data Availability

Data are contained within the article.

## Supplementary Materials

The following supporting information can be downloaded at: https://www.mdpi.com/article/10.3390/pharmaceutics14071315/s1, Figure S1: The UV–vis curves of free CPT and GT-ss-CPT conjugate.
