# Supplementary material for: Galactosed and Reduction-Responsive Nanoparticles Assembled from Trimethylchitosan–Camptothecin Conjugates for Enhanced Hepatocellular Carcinoma Therapy
Source: Pharmaceutics. 2022 Jun 21;14(7):1315. doi: 10.3390/pharmaceutics14071315 (PMC9316716; doi:10.3390/pharmaceutics14071315)
Supplement: Supplementary file 1 [file pharmaceutics-14-01315-s001.zip › pharmaceutics-1765095-supplementary.pdf]

Galactosed and Reduction-Responsive Nanoparticles Assembled from Trimethylchitosan–Camptothecin Conjugates for Enhanced Hepatocellular Carcinoma Therapy

Chen Fu [1], Jingcan Qin [2], Xinlong Liu [3] and Fei Kong [3,*]

[1] Department of Pharmacology, School of Pharmacy, China Medical University, Shenyang 110122, China; cfu@cmu.edu.cn
[2] Department of Radiology, Shanghai Jiao Tong University Affiliated Sixth People's Hospital, Shanghai Jiao Tong University School of Medicine, 600 Yi Shan Road, Shanghai 200233, China; qinjingcan1988@163.com
[3] School of Chemistry and Chemical Engineering, Shanghai Jiao Tong University, 800 Dongchuan Road, Shanghai 200240, China; xinlongliusjtu@sjtu.edu.cn
* Correspondence: kongf@sjtu.edu.cn

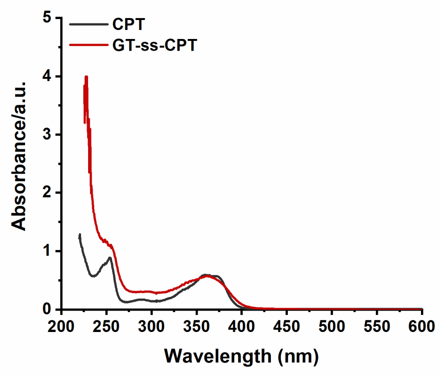

Figure S1. The UV–vis curves of free CPT and GT-ss-CPT conjugate.
